# Inflammatory Responses to Non-Typeable *Haemophilus influenzae* Clinical Isolates from Invasive and Non-Invasive Infections

**DOI:** 10.3390/pathogens14030210

**Published:** 2025-02-21

**Authors:** Brenda Huska, Marina Ulanova

**Affiliations:** Medical Sciences Division, Northern Ontario School of Medicine University, Thunder Bay, ON P7B 5E1, Canada; bmhuska@gmail.com

**Keywords:** non-typeable *Haemophilus influenzae*, inflammatory responses, macrophages, ICAM-1, TNF-α, IL-1β, lipooligosaccharide

## Abstract

Non-typeable *Haemophilus influenzae* (NTHi) is often asymptomatically carried in the upper airways but can cause a wide spectrum of disease conditions, from local respiratory tract infections to invasive disease such as sepsis or meningitis. The factors driving NTHi’s transition from benign carriage to severe systemic disease remain poorly understood. It is unknown whether NTHi strains associated with invasive or non-invasive disease differ in their capacity to trigger inflammatory responses in innate immune cells. To address this question, we have used an in vitro infection model of human THP-1 cells differentiated to macrophages. To evaluate inflammatory responses, we studied the expression of 3 prototypic pro-inflammatory molecules, ICAM-1, TNF-α, and IL-1β. The role of lipooligosaccharide in triggering inflammatory responses was assessed using inhibition of Toll-like receptor 4 signaling. Our experiments demonstrated that NTHi strains isolated from cases of invasive and non-invasive infections were similarly able to induce strong activations of macrophage pro-inflammatory responses. Our findings support the hypothesis that the development of invasive versus non-invasive NTHi disease may be more significantly influenced by the adaptive immune response than the innate immune response.

## 1. Introduction

Non-encapsulated, or non-typeable, *H. influenzae* (NTHi) is a human-restricted Gram-negative bacterium, which is commonly carried in the upper airways and considered to be a part of the normal respiratory tract microbiota [1,2]. Although these bacteria do not have the polysaccharide capsule, which is the major virulence factor of *H. influenzae,* they retain considerable pathogenic potential. Indeed, NTHi can cause a wide spectrum of clinical presentations, from sinusitis or otitis media to severe invasive disease [2]. During the past 2 decades, an increasing incidence of invasive disease caused by NTHi, such as sepsis, meningitis, or bacteremic pneumonia, has been reported worldwide [3,4,5].

In Northwestern Ontario (Canada), NTHi has been a major cause of invasive disease since 2011; extremely severe clinical presentations, including complicated pneumonia, respiratory failure, septic shock, and necrotizing fasciitis, with high case-fatality rates have been reported [6]. Yet, our recent analysis of all *H. influenzae* clinical isolates from this region found that the majority of NTHi are associated with local infections of mucosal surfaces [7]. Out of 218 NTHi collected from hospitalized patients, only 5 were isolated from blood (invasive), the remaining being isolated from the eye, ear, or sputum, and hence categorized as non-invasive [7]. Moreover, 52% of healthy children asymptomatically carried NTHi in their nasopharynx [8]. The factors driving NTHi’s transition from benign carriage to severe invasive disease remain poorly understood. It is particularly unclear whether strain-specific abilities to trigger inflammatory responses contribute to the development of the most severe forms of systemic NTHi disease.

Lipooligosaccharide (LOS) is a major virulence factor of NTHi that induces inflammatory responses via Toll-like receptor 4 (TLR4)-mediated signaling pathways [9]. In addition, other NTHi components can act as pathogen-associated molecular patterns stimulating inflammatory responses, i.e., peptidoglycan, lipoproteins, porins, unmethylated CpG dinucleotides, etc. [10,11].

Current knowledge of inflammatory responses associated with NTHi is largely based on studies of non-invasive respiratory infections. It has been established that NTHi invade airway epithelial cells and activate pro-inflammatory signaling pathways via engagement of TLR2 and TLR4, nucleotide-binding oligomerization domain-containing proteins (NOD) 1 and 2, and NOD-like receptor (NLR) inflammasomes. These pathways lead to the massive production of pro-inflammatory cytokines and chemokines by epithelial cells, including IL-1β, TNF-α, IL-6, CXCL8, and CCL2, and an increased expression of adhesion molecules such as ICAM-1. Generation of a pro-inflammatory environment in the airways leads to the recruitment of lymphocytes and innate immune cells, including neutrophils, dendritic cells, and macrophages, that in turn will result in the exaggeration of inflammatory responses. Such processes have been implicated in the pathogenesis of chronic obstructive pulmonary disease (COPD) and neutrophilic asthma and well described in the literature [12,13].

However, most published studies on inflammatory responses associated with NTHi have focused on interactions of these bacteria with respiratory epithelial cells, rather than macrophages or other innate immune cells. Yet, macrophages are essential in orchestrating inflammatory responses in the body, and analysis of their responses to NTHi strains having different invasiveness may help to clarify the reasons for the development of severe forms of this infection.

In this study, we investigated whether the pathogenicity of NTHi is linked to its ability to induce enhanced inflammatory responses in human macrophages. To address this question, we developed an experimental system, using in vitro infection of human macrophages with clinical NTHi isolates from patients with invasive or non-invasive disease. We found that NTHi strains isolated from cases of invasive and non-invasive infections were similarly able to induce strong activation of macrophage pro-inflammatory responses. Our findings suggest that macrophage-driven innate immune responses are unlikely to be a critical determinant of the clinical progression of infection.

## 2. Materials and Methods

### 2.1. Cell Culture Conditions

The methodology of this study has been previously developed in our laboratory and recently published in detail [14]. Briefly, the human THP-1 monocytic leukemia cell line (GenBank X70326, ATCC, Manassas, VA, USA) was cultured under standard conditions in RPMI-1640 medium (Sigma-Aldrich, Oakville, ON, Canada) supplemented with 20% fetal bovine serum (FBS) (R&D Systems, Inc., Minneapolis, MN, USA) and 0.4% antibiotic-antimycotic (Gibco, Eugene, OR, USA) at 37 °C and 5% CO_2_. Following 3–4 passages, when the cell number reached an approximate concentration of 1 × 10^6^ cells/mL, the cells were induced to differentiate to macrophages using 24 h long incubation in the culture medium containing 20 ng/mL phorbol myristate acetate (PMA) (Sigma-Aldrich, Oakville, ON, Canada). The procedure has been previously optimized with confirmation of differentiation to macrophages via characteristic cellular morphological changes identified by examination under microscope [14].

### 2.2. H. influenzae Strains and Culture Conditions

Non-invasive NTHi 375, isolated from middle ear of a child [15] and invasive NTHi 08-252 and 08-254 isolated from blood of pediatric patients, were provided by Dr. Raymond Tsang (National Microbiology Laboratory, Winnipeg, MB, Canada) (Table 1). Identification of *H. influenzae* was performed by standard methods and confirmed by 16S ribosomal RNA gene sequencing; PCR was performed to detect *bex*A and the serotype-specific genes [7]. Bacteria were grown on brain heart infusion (BHI) agar plates supplemented with growth factors, i.e., 10 μg/mL hemin chloride and 5 μg/mL nicotine adenine dinucleotide (NAD) as previously described [14]. For stimulation of THP-1 cells, bacterial suspensions (OD_600_ of 0.1) were prepared in BHI broth supplemented with growth factors [14].

### 2.3. Stimulation with H. influenzae

Immediately prior to stimulation, differentiated THP-1 macrophages were washed in PBS, and fresh RPMI-1640 medium supplemented with 10% FBS was added. Bacterial suspensions were used to achieve a multiplicity of infection (MOI) of 1 or 10 as previously described (14). Cell number and viability were determined with a Bright-Line Hemacytometer (Hausser Scientific, Horsham, PA, USA) using a 1:1 dilution factor with 0.4% Trypan blue solution (Sigma-Aldrich, St. Louis, MO, USA). THP-1 cells were stimulated with NTHi strains at indicated MOI, using sterile PBS for the negative control and *E. coli* lipopolysaccharide (LPS) (Invitrogen, Carlsbad, CA, USA) as the positive control. For stimulation of cells, NTHi 375 lipooligosaccharide (LOS) (provided by Dr. Andrew Cox, The National Research Council, Ottawa, ON, Canada) was also used [17]. The quantity of LOS corresponding to the number of bacterial cells used for stimulation was calculated as previously described [18]. Following adding live NTHi bacteria, LPS, or LOS, the cells were incubated at 37 °C and 5% CO_2_ for 1 h, and then 100 μg/mL gentamycin (United States Biological, Salem, MA, USA) was added. Cells were then incubated for an additional 17 h at the same incubation conditions.

In order to determine the role of LOS in cellular responses to Hia, TLR4 signaling was blocked by treating THP-1 cells with either 5 or 10 μg/mL of anti-TLR4 mouse IgG (InvivoGen) 1 h before stimulation with bacteria; to account for unspecific effects of the antibody, mouse control IgG1 (InvivoGen) was used as an isotype control. Following stimulation of THP-1 cells, supernatants were collected for use in ELISA and stored at −80 °C. Cells were collected as previously described (14) for use in flow cytometry analysis.

### 2.4. ELISA

Enzyme-linked immunosorbent assays (ELISA) were completed to quantify TNF-α and IL-1β in supernatants using uncoated ELISA kits according to the manufacturer’s instructions (Invitrogen, Carlsbad, CA, USA). Supernatants from stimulation experiments were diluted 40x to achieve a range within the limits of the assay. Samples were run in duplicate; the means of the reading of 2 wells were used for analysis. Plates were read at 450 nm and 570 nm for wavelength subtraction.

### 2.5. Flow Cytometry Analysis

To detect the cell surface expression of ICAM-1, immunostaining of THP-1 cells with 1 μg/mL phycoerythrin (PE)-conjugated mouse-antihuman ICAM-1 (BD Biosciences, Mississauga, ON, Canada) was conducted as previously described [14]. To test for cell death, cells were stained with 1 μg/mL propidium iodide (PI) (BD Biosciences) immediately prior to flow cytometry. Flow cytometry was performed on the SONY SA3800 spectral cell analyzer with SA3800 Software version 2.0.5.54250 (SONY Corporation, CA, USA). The cell population was gated based on light scattering properties; 10,000 gated events were collected as displayed in Figure S1 (Supplementary Materials). The gates for PI-negative cells were created, and PI-positive cells were excluded from the analysis (Figure S1). The results were presented as the mean fluorescence intensity (MFI) for the ICAM-1 positive, PI-negative population. To exclude non-specific antibody binding from analysis, we used PE-conjugated isotype control (Invitrogen) in the same concentration, as ICAM-1 antibody. Representative results of flow cytometry analysis are displayed in Figure S2 (Supplementary Materials).

### 2.6. Statistical Analysis

Data were expressed as a mean of at least 3 independent experiments. Statistical difference was determined with a one-way analysis of variance (ANOVA) with a Tukey post-hoc test or Student’s t-test for paired comparisons. A *p* value of < 0.05 was reported as statistically significant. Statistical analysis was performed using Graph-Pad Prism 9 (GraphPad Prism Software Inc., San Diego, CA, USA).

## 3. Results

### 3.1. Stimulation of Differentiated THP-1 Cells with Invasive or Non-Invasive Clinical NTHi Isolates Resulted in an Increase in Cell Surface Expression of ICAM-1 and in TNF-α and IL-1β Release

To determine whether the abilities of clinical NTHi isolates to induce inflammatory responses in human macrophages differ between invasive and non-invasive strains, THP-1 cells differentiated into mature macrophages were infected in vitro with three different NTHi strains of different pathogenicity, isolated from patients with invasive or non-invasive disease (Table 1). Following stimulation, macrophages were collected, and cell surface expression of intercellular adhesion molecule 1 (ICAM-1, aka CD54), a key pro-inflammatory and co-stimulatory molecule, which serves as a receptor for NTHi [19], was studied using immunostaining and flow cytometry analysis. The concentrations of pro-inflammatory cytokines TNF-α and IL-1β in the supernatants were measured using ELISA.

**Figure 1:**
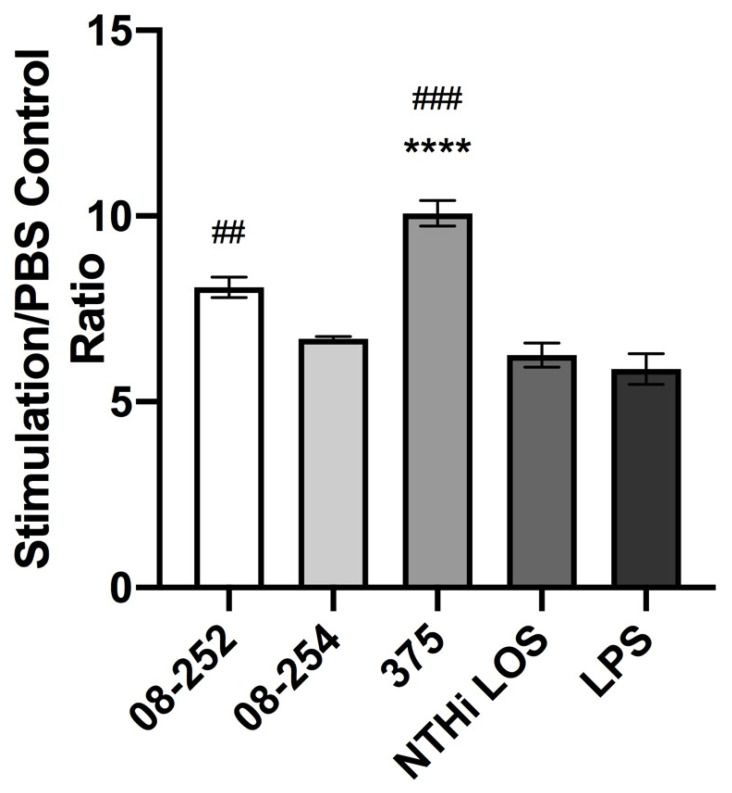
Cell surface expression of ICAM-1 in differentiated THP-1 cells stimulated with non-typeable *H. influenzae* (NTHi) clinical isolates 08-252 (*n* = 4), 08-254 (*n* = 3), and 375 (*n* = 3) at MOl 10, NTHi 375 LOS at 48 ng/mL (*n* = 3), or *E. coli* LPS at 100 ng/mL (*n* = 9) for 18 h as described in Methods (flow cytometry analysis). **** *p* < 0.0001 statistical significance between treatments and LOS; ^##^ *p* < 0.01 and ^###^ *p* < 0.001, statistical significance between treatments and LPS; MOI, multiplicity of infection; stimulation/PBS control ratio, Mean ± SD.

Stimulation of differentiated THP-1 cells with three NTHi strains at MOI 10 resulted in 6–10-fold increase in ICAM-1 expression as compared to PBS control. The highest increase was induced by the stimulation with non-invasive NTHi 375 (10-fold increase) (Table 2, Figure 1). Under the same experimental conditions, NTHi 375 LOS used at a concentration of 48 ng/mL that was equivalent to the amount of LOS present in 5 × 10^6^ bacterial cells (MOI 10) induced significantly lower ICAM-1 expression than whole NTHi 375 bacterial cells (*p* < 0.0001) (Table 2, Figure 1). 

**Figure 2:**
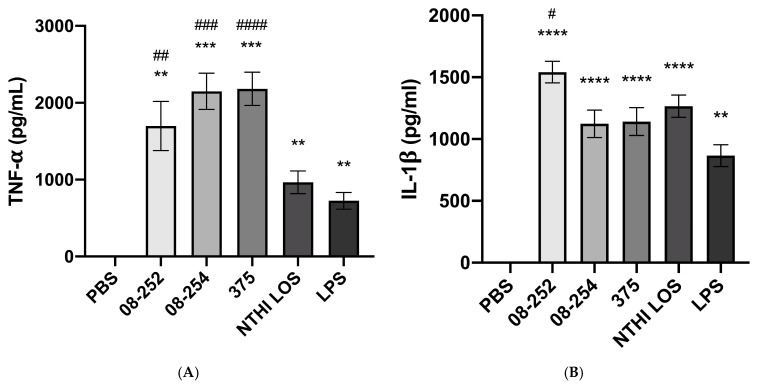
Cytokine release from differentiated THP-1 cells stimulated with non-typeable *H. influenzae* (NTHi) clinical isolates 08-252 (*n* = 4), 08-254 (*n* = 8), and 375 (*n* = 7) at MOl 10, NTHi LOS at 48 ng/mL (*n* = 3), or *E. coli* LPS at 100 ng/mL (*n* = 7) for 18 h as described in Methods. Concentrations of TNF-α (**A**) and IL-1β (**B**) were measured by ELISA (pg/mL). Data represent mean cytokine concentrations ± SEM. ** *p* < 0.01, *** *p* < 0.001, and **** *p* < 0.0001, statistical significance between treatments and PBS; ^#^ *p* < 0.05, ^##^ *p* < 0.1, ^###^ *p* < 0.001, and ^####^ *p* < 0.0001, between bacterial stimulation and LPS.

Under the same conditions of stimulation, significant release of pro-inflammatory cytokines TNF-α and IL-1β into cell culture supernatants was induced by all three NTHi strains as well as by NTHi 375 LOS and *E. coli* LPS (Figure 2A,B). No significant difference in TNF-α induced by different NTHi strains was found (Figure 2A); the level of IL-1β was higher following stimulation with NTHi 08-252 compared to NTHi 08-254 and NTHi 375 (*p* < 0.05) (Figure 2B). Isolated NTHi 375 LOS induced lower TNF-α release than whole NTHi 375 (*p* < 0.05). In contrast, the release of IL-1β induced by isolated LOS was comparable to the effect of whole bacteria, *p* > 0.05 (Figure 2A,B).

### 3.2. Immunostimulatory Effect of Hia Partially Depended on LOS

**Figure 3:**
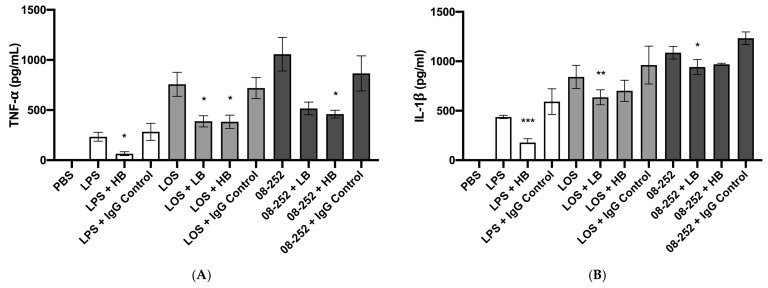
Cytokine release from differentiated THP-1 cells stimulated with non-typeable *H. influenzae* (NTHi) clinical isolate 08-252 at MOl 1, *E. coli* LPS, or NTHi LOS at 10 ng/mL for 18 h following inhibition of TLR4 as described in Methods. Concentrations of TNF-α (**A**) and IL-1β (**B**) were measured by ELISA (pg/mL). THP-1 cells were pre-treated with 5 (LB) or 10 μg/mL (HB) mouse anti-TLR4 IgG 1 h prior to stimulation. IgG Control, mouse control IgG1 at concentration of 10 μg/mL; * *p* < 0.05, ** *p* < 0.01, and *** *p* < 0.001, statistical significance between treatments and IgG control. Data represent mean cytokine concentrations ± SEM (*n* = 4 independent experiments).

Previous studies suggested that some specific LOS modifications are more prevalent among NTHi causing invasive than non-invasive disease [20]. To determine to which degree the invasive capabilities of NTHi depend on the engagement of TLR4 with LOS, we inhibited TLR4 using pre-treatment of THP-1 cells with anti-TLR4 mouse IgG prior to infection, with mouse IgG1 serving as an isotype control. These experiments involved extensive optimization as we have tested multiple combinations of anti-TLR4 treatment procedures with different MOI and LOS concentrations to achieve the best possible balance between the strength of cellular stimulation and the amount of blocking antibody. We have found out that stronger stimulation was associated with a smaller blocking effect. Therefore, in these experiments we used a MOI 1 and LOS concentration of 10 ng/mL (Figure 3A,B). The anti-TLR used in either 5 or 10 μg/mL concentration had a significant inhibitory effect on TNF-α induced by NTHi LOS (*p* < 0.05) (Figure 3A). The anti-TLR4 used at a high concentration significantly inhibited TNF-α release induced by the invasive NTHi strain 08-252 as compared to the isotype control (*p* < 0.05); at low concentration, the inhibitory effect was noted, but it did not reach statistical significance (*p* > 0.05) (Figure 3A). The effect of *E. coli* LPS on TNF-α release was also inhibited by anti-TLR4 at 10 μg/mL (*p* < 0.05) (Figure 3A).

Under the same conditions, we observed the inhibitory effect of anti-TLR4 on IL-1β release by invasive NTHi- or LOS-stimulated cells (*p* < 0.05 and *p* < 0.01, respectively), although it appeared smaller compared to TNF-α release; inhibition of TLR4 also decreased the release of IL-1β induced by LPS (*p* < 0.001) (Figure 3B). The isotype control antibody did not decrease TNF-α or IL-1β release induced by any treatment (Figure 3A,B).

## 4. Discussion

Although NTHi is best known for its propensity to cause local (non-invasive) infections of the respiratory tract and otitis media, over the past 2 decades, an increasing incidence of invasive NTHi disease has been reported worldwide, with the majority of cases occurring in infants, the elderly, and immunocompromised individuals [2,3]. NTHi has also been recognized as a significant cause of invasive perinatal infections with high fatality rates [21]. There is no specific vaccine available to prevent NTHi infections; the development of such a vaccine met significant challenges due to a great genetic variability of clinical NTHi isolates and an incomplete understanding of mechanisms of protective immunity against this pathogen [22]. NTHi engage in highly complex interactions with the host immune system, driven by the presence of multiple virulence factors—some with phase-variable expression—and their extensive capacity for immune evasion [23]. Previous studies have found that NTHi isolates from invasive disease differ phenotypically and genetically from non-invasive strains which cause otitis media or are asymptomatically carried in the nasopharynx [24]. However, invasive isolates are also heterogeneous, exhibiting significant differences in genotype and resistance to the bactericidal effects of human serum [20,25,26,27].

The ability of NTHi to activate innate immune and inflammatory responses has been extensively studied in models of non-invasive diseases, such as bronchial and ear infections, with a primary focus on epithelial cells [28,29]. Mechanisms of inflammatory responses triggered by NTHi adhesion to and invasion of epithelial cells have been analysed; the findings contributed to understanding of the pathogenesis of non-invasive NTHi disease [30]. Pro-inflammatory responses of macrophages infected with non-invasive NTHi have also been demonstrated [18,31,32]. Certain structural LOS characteristics, resulting from phase variation in specific LOS biosynthetic genes, have been linked to NTHi’s ability to cause invasive versus non-invasive disease [33]. Although recent studies elucidated the role of diverse bacterial virulence factors in host–pathogen interactions [23], the pathogenic mechanisms underlying invasive NTHi disease are not yet fully understood. It is still unclear whether NTHi strains associated with invasive or non-invasive disease differ in their capacity to trigger inflammatory responses in innate immune cells. To address this question, we have used an in vitro infection model of human THP-1 cells differentiated to macrophages. To evaluate inflammatory responses, we studied the expression of three prototypic pro-inflammatory molecules, ICAM-1, TNF-α, and IL-1β.

The adhesion molecule ICAM-1 is a transmembrane glycoprotein, member of the immunoglobulin superfamily, which enables critical intercellular interactions in the process of leukocyte trafficking during inflammatory responses [34]. In addition, ICAM-1 expressed on antigen-presenting cells, such as macrophages, is involved in the process of immunological synapse formation via interaction with its counterreceptor LFA-1 on T cells. Through this interaction, ICAM-1 delivers co-stimulatory signals necessary for T-cell activation, hence ICAM-1 represents a critical molecule involved in both innate and adaptive immune responses [34]. It has been established that ICAM-1 expressed on airway epithelial cells acts as a receptor for *H. influenzae*, enabling bacterial adhesion through interaction with the outer membrane protein P5 and the Type IV pilus of NTHi [19,35]. However, the role of ICAM-1 in interactions of *H. influenzae* with innate immune cells, such as macrophages, remains unclear, and it is unknown if any specific bacterial structures are involved in such interactions. In the context of bacterial infections, most published studies have focused on ICAM-1 expression in epithelial or endothelial cells, rather than in leukocytes [34].

In our experiments, the cell surface expression of ICAM-1 significantly increased after infection with both invasive and non-invasive NTHi isolates, demonstrating an activation of macrophages that may enhance their functions. In addition, NTHi infection with any strain induced a significant release of TNF-α and IL-1β, further supporting cellular activation. The production of both cytokines and ICAM-1 is regulated by the transcription factor NF-κB, following pro-inflammatory signaling initiated by the recognition of pathogen-associated molecular patterns (PAMP) by Pattern Recognition Receptors (PRR) [36]. LOS is a prominent PAMP and the major virulence factor of both encapsulated and non-encapsulated *H. influenzae.* Unlike LPS typical for most Gram-negative bacteria, LOS lacks the repeating O-antigen but includes the tri-heptose oligosaccharide backbone covalently attached to a 3-deoxy-d-manno-oct-2-ulosonic acid moiety (KDO), that is the core region [9]. The core region is covalently linked to the lipid A, a TLR4 ligand, which has powerful abilities to activate innate immune and inflammatory responses [37]. In our experiments, exposure of THP-1 cells to a neutralizing TLR4 antibody [38] led to a reduction in TNF-α and IL-1β production induced by an invasive NTHi isolate, highlighting the importance of TLR4-mediated signaling in macrophage pro-inflammatory responses to NTHi. Although TLR4 inhibition did not entirely abolish macrophage responses, it more effectively suppressed TNF-α release compared to IL-1β release.

In contrast to TNF-α production, which is directly induced by TLR4 downstream signaling leading to the activation and nuclear translocation of NF-κB [39], IL-1β production relies on a two-signal mechanism [40]. The primary signal, largely mediated by the NF-κB activation, leads to the expression of pro-IL-1β; yet the secondary signal is required for the activation of the inflammasome complex, being an essential factor for mature IL-1β production [41]. The ability of *H. influenzae* to activate the NLRP3 inflammasome has been demonstrated in an in vitro model of NTHi infection [42]. NTHi express a plethora of immunostimulatory molecules recognized by innate immune receptors including those implicated in the inflammasome formation [43]. Given that inhibition of TLR4 did not completely abrogate TNF-α or IL-1β release following bacterial stimulation, the role of NTHi immunostimulatory molecules beyond LOS could be significant. The immunostimulatory effect of *H. influenzae* could be mediated by the recognition of peptidoglycan, porin, and DNA, by NOD1, TLR2, and TLR9, correspondingly [44,45,46]. In our in vitro model, we used cellular stimulation with live NTHi followed by incubation with killed bacteria that may deliver a strong RNA signal to activate PRRs. Prokaryotic RNA has previously been identified as a particular class of viability-associated PAMPs [47].

Our experiments demonstrated that NTHi isolates from clinical cases of both invasive and non-invasive infections are similarly able to induce strong activation of macrophage pro-inflammatory responses. Such responses are essential in host defenses against bacterial infections. Activated macrophages are potent phagocytic cells capable to effectively destroy engulfed bacteria and present their antigens to T lymphocytes. Moreover, macrophages are crucial in facilitating adaptive immune responses due to their ability to activate T- and B-lymphocytes through both cell-associated co-stimulatory molecules, such as ICAM-1, and soluble signals, such as cytokines [48]. Our study suggests that the progression to invasive versus non-invasive NTHi disease is unlikely determined by the macrophages’ ability to mount pro-inflammatory responses following bacterial exposure. The transition of NTHi from benign airway colonization to bloodstream invasion may primarily depend on the mucosal immune system’s ability to prevent breaches of the epithelial barriers and, ultimately, on the adaptive immune system’s capacity to control replicating bacteria entering the bloodstream. The importance of the mucosal immune system in defending against NTHi has been highlighted by studies examining the roles of B cells and secretory IgA in airway infection associated with COPD [49,50,51]. Moreover, it was demonstrated that serum IgG and IgM LOS-specific antibodies exhibited bactericidal activity against NTHi [17]. Recent studies elucidated the role of adaptive immune responses in protection against invasive NTHi infections; specific biological characteristics were found to be associated with the ability of NTHi to cause invasive rather than non-invasive disease [25,27]. It was discovered that the rapid and reversible on-off switching of gene expression (phase-variation) of LOS glycosyltransferases occurred during transition from non-invasive to invasive NTHi infection [20]. Because of these changes in gene expression, which led to alterations in LOS structure, NTHi became more resistant to the bactericidal effect of human serum [20]. It was also demonstrated that invasive NTHi isolates were more resistant to complement-mediated killing than non-invasive NTHi due to an enhanced ability of invasive bacteria to evade IgM binding [26]. Indeed, it has been demonstrated that the development of invasive NTHi disease is associated with lack of circulating IgG and IgM antibody specific to NTHi antigens. These antibodies are essential for opsonizing bacteria for phagocytosis and killing bacteria following the classical complement activation with formation of the terminal membrane attack complex. In our earlier studies, we found that patients with secondary immunodeficiency states due to hematological malignancies and end-stage renal disease had decreased serum antibody levels to protein D, one of the well-characterized protective NTHi antigens included in a pediatric vaccine Synflorix [52]. In addition, epidemiological studies indicated that invasive NTHi disease is strongly associated with insufficient humoral immunity, such as lack of natural antibodies in the sera of newborns and infants or in the elderly due to age-dependent B-cell function decline, as well as in individuals with hypogammaglobulinemia or other primary and secondary immunodeficiency states [53,54,55].

Interestingly, NTHi 375, a non-invasive strain isolated from ear effusion, appeared to be able to induce higher expression of ICAM-1 in THP-1 cells compared to two invasive NTHi strains. Considering that ICAM-1 acts as a receptor facilitating NTHi invasion of epithelial cells [19,35], these observations suggest the adaptation of this strain to the environment. Indeed, the enhanced abilities of NTHi to adhere to host cells may contribute to the pathogenesis of mucosal infections; these features might be selected as a result of bacterial competition for a specific ecological niche. Although infection with two invasive NTHi strains did not cause noticeable differences in ICAM-1 or TNF-α expression, NTHi 08-252 induced higher IL-1β release. Factors, which cause increased capacities to activate some parts of complicated pathways leading to IL-1β production, require further mechanistic studies.

This study has some limitations. During the in vivo infectious process, multiple innate immune cells, including dendritic cells, neutrophils, and NK cells, in addition to macrophages, can be important in determining the outcome of an NTHi infection. In particular, neutrophils are essential in host defenses due to their capacities to engulf and kill phagocytosed bacteria and capture bacteria for degradation via the release of neutrophil extracellular traps (NETs) [27]. The use of an in vitro model of bacterial infection inevitably underestimates complex interactions among various innate immune cells and between innate and adaptive immune mechanisms. Moreover in vivo, NTHi are able to form biofilms, which may interfere with their interaction with host cells, that can result in evading immune responses [23]. Although a reductionistic approach helps to address some specific questions and the THP-1 cell line differentiated to macrophages is widely used in many in vitro studies, this model does not completely replicate the response of human macrophages to bacterial infection. Further studies on primary human monocytic cells would help to clarify the role of innate immune cell activation in NTHi pathogenesis. To advance our understanding of the true pathophysiology of NTHi infections, it will be important to conduct experiments involving the formation of biofilms, to get insight into the role of this pathogenetic mechanism in determining the outcomes of the infectious process.

While we selected three clinical isolates on the base of their distinct features, they do not represent the whole spectrum of NTHi, characterized by notable genetic heterogeneity.

## 5. Conclusions

Our findings suggest that NTHi’s ability to activate inflammatory responses is not the critical factor in determining the progression to invasive versus non-invasive disease. However, strong activation of inflammatory responses exhibited by NTHi-infected macrophages can be significant for host defenses as innate immune cells provide activation signals to adaptive immunity. Adaptive immune responses can be critical in determining the outcomes of the infectious process, from upper respiratory tract colonization to severe systemic disease. The development of invasive versus non-invasive clinical disease may depend on the adaptive immune system’s ability to control rapidly replicating bacteria through antibodies targeting specific NTHi antigens, which then opsonize bacteria for phagocytosis. Insufficient adaptive immune responses likely drive increased susceptibility to invasive NTHi infections in newborns, the elderly, and immunocompromised individuals. Although creating a vaccine against NTHi meets significant challenges, immunization with protective NTHi antigens, such as LOS, outer membrane proteins, and adhesins, represents a promising strategy to prevent severe invasive disease in vulnerable population groups.

## Figures and Tables

**Table 1:** Characteristics of non-typeable *H. influenzae* (NTHi) strains.

Label	Site of Isolation	Invasiveness	Sequence Type (ST) ^1^	Notes
08-252	Blood	yes	389	Source: Dr. Tsang’s collection
08-254	Blood	yes	599	Source: Dr. Tsang’s collection
375	Middle ear effusion	no	3	Described in [15]

^1^ Determined by the multilocus sequence typing [16].

**Table 2:** Flow cytometry analysis of ICAM-1 cell surface expression in differentiated THP-1 cells stimulated with non-typeable *H. influenzae* (Mean ± SD).

Stimulation	MFI	Stimulation/PBS Control Ratio
*H. influenzae* NTHi 08-252	96,730.5 ± 6538	8.08 ± 0.55
*H. influenzae* NTHi 08-254	59,459.4 ± 10,027	6.69 ± 0.10
*H. influenzae* NTHi 375	59,768.5 ± 9427	10.07 ± 0.60 **
NTHi LOS	77,743.5 ± 6827	6.26 ± 0.56
*E. coli* LPS	73,447.7 ± 6538	5.88 ± 1.22

MFI, mean fluorescence intensity; LOS, lipooligosaccharide; LPS, lipopolysaccharide, ** *p* < 0.01, NTHi 375 vs. NTHi 08-254.

## Data Availability

The data presented in this study are contained within the article or Supplementary Materials.

## Supplementary Materials

The following supporting information can be downloaded at https://www.mdpi.com/article/10.3390/pathogens14030210/s1; Figure S1: flow cytometry light scattering (forward scatter, FSC, and side scatter, SSC) dot plot of a representative experiment showing gating of a total desired cell population (**A**), gating of PI-negative cells within the gated population A (**B**), and a histogram of PI-negative ICAM-1-positive cells (**C**) and PI-positive cells (**D**) with PE meaning phycoerythrin and PI meaning propidium iodine; Figure S2: cell surface expression of ICAM-1 on differentiated THP-1 cells stimulated with non-typeable *H. influenzae* (NTHi) clinical isolates 08-252, 08-254, and 375 at MOl 10, NTHi 375 LOS at 48 ng/mL, or *E. coli* LPS at 100 ng/mL for 18 h as described in Methods (flow cytometry analysis). Histograms show ICAM-1 positive events in the PI-negative gate (results of one representative experiment) with PE meaning phycoerythrin.
