# Supplementary material for: Inflammatory Responses to Non-Typeable Haemophilus influenzae Clinical Isolates from Invasive and Non-Invasive Infections
Source: Pathogens. 2025 Feb 21;14(3):210. doi: 10.3390/pathogens14030210 (PMC11945879; doi:10.3390/pathogens14030210)
Supplement: Supplementary file 1 [file pathogens-14-00210-s001.zip › pathogens-3463114-figures S1.pdf]

Figure S1

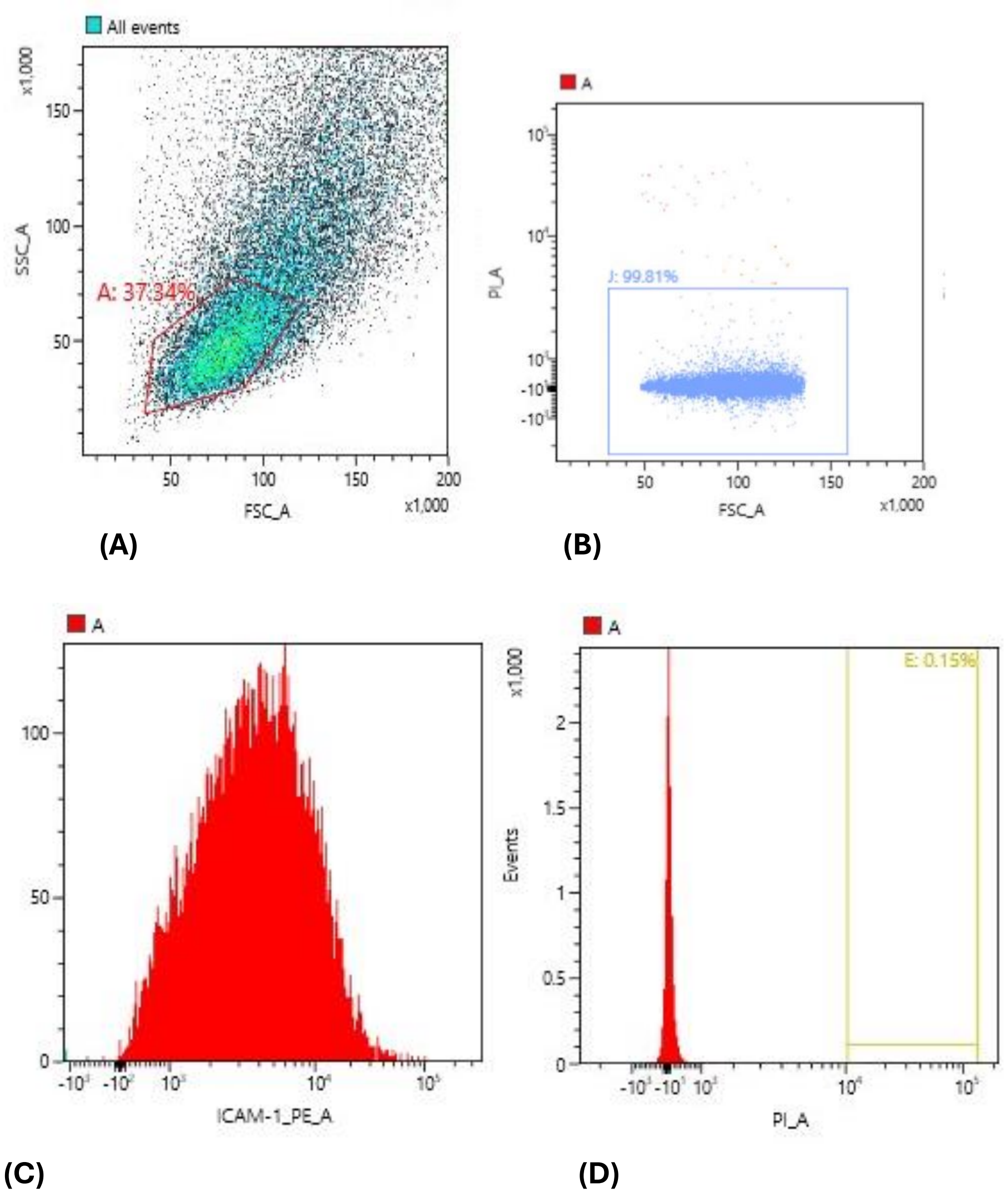

Flow cytometry light scattering (forward scatter, FSC, side scatter, SSC) dot-plot of a representative experiment showing gating of a total desired cell population (A); gating of PI-negative cells within the gated population A (B); a histogram of PI-negative ICAM-1-positive cells (C) and PI-positive cells (D). PE, phycoerythrin, PI, propidium iodine.
