# Supplementary material for: Inflammatory Responses to Non-Typeable Haemophilus influenzae Clinical Isolates from Invasive and Non-Invasive Infections
Source: Pathogens. 2025 Feb 21;14(3):210. doi: 10.3390/pathogens14030210 (PMC11945879; doi:10.3390/pathogens14030210)
Supplement: Supplementary file 1 [file pathogens-14-00210-s001.zip › pathogens-3463114-supplementary S2.pdf]

Figure S2

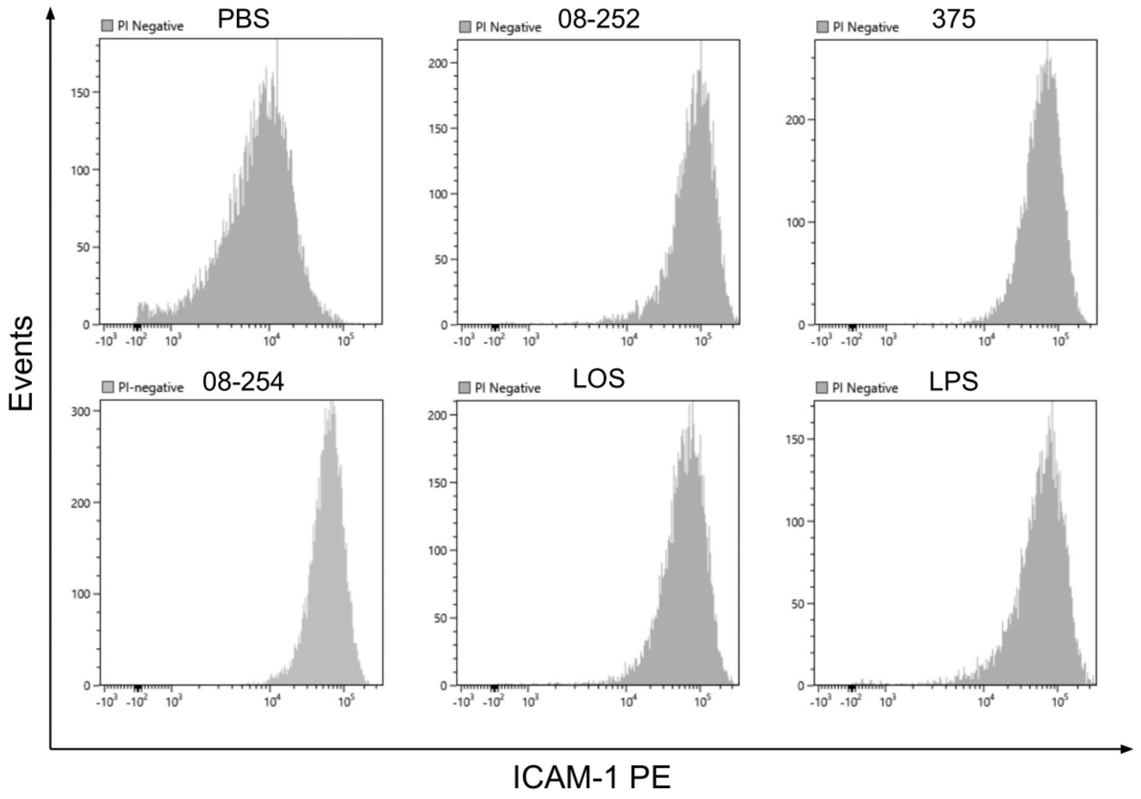

Cell surface expression of ICAM-1 on differentiated THP-1 cells stimulated with non-typeable *H. influenzae* (NTHi) clinical isolates 08-252, 08-254, 375 at MOI 10, NTHi 375 LOS at 48 ng/mL, or *E. coli* LPS at 100 ng/ml for 18 hours as described in Methods (flow cytometry analysis). Histograms show ICAM-1 positive events in the PI-negative gate (results of one representative experiment). PE, phycoerythrin.
